# Target product profiles for paediatric formulations of azithromycin and nitrofurantoin

**DOI:** 10.2471/BLT.25.294422

**Published:** 2025-11-03

**Authors:** Yasir Bin Nisar, Giulia Brigadoi, Shabina Ariff, Narendra Kumar Arora, Adrie Bekker, James A Berkley, Julia Anna Bielicki, Tim R Cressey, Devika Dixit, Lisa Frigati, Amanda Gwee, Manji Karim, Rajiv Kshirsagar, Hilda A Mujuru, Victor Musiime, Mary Ojoo, Shalini Sri Ranganathan, Emmanuel Roilides, Michael Sharland, Catherine Tuleu, Robinson D Wammanda, Phoebe Williams, Martina Penazzato

**Affiliations:** a Department of Sexual, Reproductive, Maternal, Child and Adolescent Health and Ageing, World Health Organization, Avenue Appia 20, 1211 Geneva 27, Switzerland.; b Department of Women’s and Children’s Health, Padua University Hospital, Padua, Italy.; c Department of Pediatrics & Child Health, Aga Khan University Hospital, Karachi, Pakistan.; d INCLEN Trust International, New Delhi, India.; e Family Centre for Research with Ubuntu, Stellenbosch University, Cape Town, South Africa.; f Childhood Acute Illness and Contribution Network, Nairobi, Kenya.; g Faculty of Medicine, University of Basel, Basel, Switzerland.; h Faculty of Associated Medical Sciences, Chiang Mai University, Chiang Mai, Thailand.; i Department of Pediatrics, University of Calgary, Calgary, Canada.; j Murdoch Children’s Research Institute, Melbourne, Australia.; k Department of Pediatrics and Child Health, Muhimbili University of Health and Allied Sciences, Dar es Salaam, United Republic of Tanzania.; l Medicines and Nutrition Center, UNICEF Supply Division, Copenhagen, Denmark.; m Department of Child, Adolescent and Women Health, University of Zimbabwe, Harare, Zimbabwe.; n College of Health Sciences, Makerere University, Kampala, Uganda.; o Faculty of Medicine, University of Colombo, Colombo, Sri Lanka.; p Aristotle University School of Medicine, Thessaloniki, Greece.; q Institute for Infection and Immunity, St George’s University of London, London, England.; r School of Pharmacy, University College London, London, England.; s Ahmadu Bello University Teaching Hospital, Zaria, Nigeria.; t Faculty of Medicine, University of Sydney, Sydney, Australia.; u Science for Health Department, World Health Organization, Geneva, Switzerland.

## Abstract

Bacterial infections are still a main cause of death in children younger than 5 years, yet few age-appropriate antibiotic formulations exist, which limits treatment options and compromises quality of care. In 2023, the World Health Organization (WHO) published its first list of priority paediatric antibiotic formulations to guide research and development for age-appropriate antibiotic formulations. Both azithromycin and nitrofurantoin are on this list. Currently, no dispersible tablets are approved or available for these drugs and existing liquid forms are poorly palatable and/or contain excipients of safety concern. To support the development of age-appropriate formulations for these two antibiotics, we produced target product profiles using WHO’s methods. For azithromycin, the optimum age-appropriate formulation and dose is scored 100 mg dispersible tablets or an orodispersible 50 mg multiparticulate formulation, with dispersible 50 mg tablets as the minimum requirement. For nitrofurantoin, the optimum age-appropriate formulation is an orodispersible multiparticulate formulation or scored dispersible tablets, with dispersible tablets as the minimum requirement. Based on the WHO recommended dosage of 4 mg/kg per day for children for nitrofurantoin, the optimum unit dose is 5 mg. If scoring is feasible, a 10 mg unit dose should be developed for dosing flexibility across paediatric age groups. These profiles aim to support regulatory authorities, pharmaceutical developers, health programmes and other stakeholders in advancing safer, effective and child-appropriate antibiotic formulations.

## Résumé

Les infections bactériennes restent une cause majeure de mortalité chez les enfants de moins de 5 ans, mais il existe peu de formulations antibiotiques adaptées à leur âge, ce qui limite les options thérapeutiques et compromet la qualité des soins. En 2023, l’Organisation mondiale de la Santé (OMS) a publié sa première liste de formulations antibiotiques pédiatriques prioritaires afin d’orienter la recherche et le développement de formulations antibiotiques adaptées à l’âge. L’azithromycine et la nitrofurantoïne figurent toutes deux sur cette liste. À l’heure actuelle, aucun comprimé dispersible n’est approuvé ou disponible pour ces médicaments, et les formes liquides existantes sont peu agréables au goût et/ou contiennent des excipients qui soulèvent des questions de sécurité. Afin de soutenir le développement de formulations adaptées à l’âge pour ces deux antibiotiques, nous avons établi des profils de produits cibles en appliquant les méthodes de l’OMS. Pour l’azithromycine, la formulation et la posologie optimales adaptées à l’âge sont des comprimés dispersibles de 100 mg ou une formulation multiparticulaire orodispersible de 50 mg, avec des comprimés dispersibles de 50 mg comme exigence minimale. Pour la nitrofurantoïne, la formulation optimale adaptée à l’âge est une formulation multiparticulaire orodispersible ou des comprimés dispersibles sécables, avec des comprimés dispersibles comme exigence minimale. Sur la base de la posologie recommandée par l’OMS de 4 mg/kg par jour pour les enfants pour la nitrofurantoïne, la dose unitaire optimale est de 5 mg. S’il est possible de produire des comprimés sécables, une dose unitaire de 10 mg devrait être mise au point pour permettre une flexibilité dans le dosage pour tous les groupes d’âge pédiatriques. Ces profils visent à aider les autorités réglementaires, les concepteurs de produits pharmaceutiques, les programmes de santé et d’autres parties prenantes à promouvoir des formulations antibiotiques plus sûres, efficaces et adaptées aux enfants.

## Resumen

Las infecciones bacterianas siguen siendo una de las principales causas de mortalidad en niños menores de 5 años; sin embargo, existen pocas formulaciones antibióticas adecuadas para la edad, lo que limita las opciones terapéuticas y compromete la calidad de la atención. En 2023, la Organización Mundial de la Salud (OMS) publicó su primera lista de formulaciones antibióticas pediátricas prioritarias para orientar la investigación y el desarrollo de formulaciones adecuadas a la edad. Tanto la azitromicina como la nitrofurantoína figuran en esa lista. Actualmente, no existen comprimidos dispersables aprobados o disponibles para estos medicamentos. Además, las formas líquidas existentes presentan mal sabor o contienen excipientes que plantean preocupaciones de seguridad. Para respaldar el desarrollo de formulaciones adecuadas para la edad de estos dos antibióticos, se elaboraron perfiles de producto objetivo utilizando los métodos de la OMS. En el caso de la azitromicina, la formulación y dosis óptimas adecuadas para la edad corresponden a comprimidos dispersables ranurados de 100 mg o una formulación multiparticulada orodispersable de 50 mg, siendo los comprimidos dispersables de 50 mg el requisito mínimo. Para la nitrofurantoína, la formulación óptima adecuada para la edad es una formulación multiparticulada orodispersable o comprimidos dispersables ranurados, siendo los comprimidos dispersables el requisito mínimo. Con base en la dosis recomendada por la OMS de 4 mg/kg por día para niños, la dosis unitaria óptima de nitrofurantoína es de 5 mg. Si resulta viable el ranurado, debería desarrollarse una dosis unitaria de 10 mg para ofrecer flexibilidad en la dosificación entre los distintos grupos de edad pediátrica. El objetivo de estos perfiles es apoyar a las autoridades reguladoras, los desarrolladores farmacéuticos, los programas de salud y otros interesados en el avance de formulaciones antibióticas más seguras, eficaces y adecuadas para la población infantil.

## ملخص

لا تزال العدوى البكتيرية سببًا رئيسيًا للوفاة لدى الأطفال دون سن الخامسة، ومع ذلك، لا تتوفر سوى تركيبات قليلة من المضادات الحيوية المناسبة لأعمارهم، مما يحد من خيارات العلاج ويؤثر سلبًا على جودة الرعاية. في عام 2023، نشرت منظمة الصحة العالمية (WHO) أول قائمة لها بتركيبات المضادات الحيوية ذات الأولوية للأطفال لتوجيه البحث والتطوير لإيجاد تركيبات مناسبة لأعمارهم. ويوجد كل من أزيثروميسين ونيتروفورانتوين في هذه القائمة. حاليًا، لا توجد أقراص قابلة للتشتت معتمدة أو متوفرة لهذين الدواءين، والأشكال السائلة الحالية غير مستساغة و/أو تحتوي على سواغات تُثير مخاوف بشأن السلامة. لدعم تطوير تركيبات مناسبة للعمر لهذين المضادين الحيويين، قمنا بإعداد ملفات تعريف المنتجات المستهدفة باستخدام أساليب منظمة الصحة العالمية. بالنسبة لأزيثروميسين، تُعدّ التركيبة والجرعة المناسبة للعمر هي أقراص قابلة للانتشار بتركيز 100 ملج، أو تركيبة فموية متعددة الجسيمات قابلة للانتشار، مع الحد الأدنى المطلوب لأقراص قابلة للانتشار بتركيز 50 ملج. أما بالنسبة للنيتروفورانتوين، فتعد التركيبة المناسبة للعمر هي تركيبة فموية متعددة الجسيمات قابلة للانتشار، أو أقراص قابلة للانتشار بتركيز 50 ملج. بناءً على الجرعة الموصى بها من جانب منظمة الصحة العالمية (WHO) للأطفال من النيتروفورانتوين، والبالغة 4 ملج/كج يوميًا، فإن الجرعة المثلى هي 5 ملج. إذا كان التقييم ممكنًا، فينبغي تطوير جرعة 10 ملج لمرونة الجرعات بين الفئات العمرية المختلفة للأطفال. تهدف هذه الملفات التعريفية إلى دعم الجهات التنظيمية، ومطوري الأدوية، والبرامج الصحية، والجهات المعنية الأخرى في تطوير تركيبات مضادات حيوية أكثر أمانًا وفعالية ومناسبة للأطفال.

## 摘要

细菌感染目前仍然是 5 岁以下儿童的主要死因之一，但是适用于幼龄儿童的抗生素制剂却寥寥无几，这导致治疗选择受限且护理质量大打折扣。世卫组织 (WHO) 于 2023 年发布了首份重点儿科抗生素制剂清单，以为适用于幼龄儿童的抗生素制剂的研发工作提供指导。阿奇霉素和呋喃妥因均在该清单之列。目前，市面上尚无任何获批或可用的阿奇霉素和呋喃妥因分散片，且现有液体剂型口感欠佳和/或含有存在安全问题的辅料。为了支持研发这两种适用于幼龄儿童的抗生素制剂，我们运用世卫组织的相关方法编写了目标产品概况。适用于幼龄儿童的阿奇霉素的最佳制剂和剂量是带有刻痕的 100 毫克分散片或口分散多颗粒制剂，且要求以 50 毫克分散片作为最低制剂和剂量标准。适用于幼龄儿童的呋喃妥因的最佳制剂是口分散多颗粒制剂或带有刻痕的分散片，且要求以分散片作为最低制剂标准。鉴于世卫组织建议每日按 4 毫克/千克的标准确定儿童的呋喃妥因使用剂量，其最佳单位剂量为 5 毫克。如果刻痕技术切实可行，则应研发单位剂量为 10 毫克的分散片，以便在各儿科年龄组之间灵活调整用量。此类产品概况旨在为监管机构、药品开发商、卫生方案制定机构和其他利益攸关方提供支持，以推动研发更加安全有效且适用于儿童的抗生素制剂。

## Резюме

Бактериальные инфекции по-прежнему являются основной причиной смертности среди детей в возрасте до 5 лет, при этом антибиотических препаратов в формах, соответствующих возрасту пациентов, все еще мало, что ограничивает возможные варианты лечения и приводит к ухудшению качества медицинской помощи. В 2023 году Всемирная организация здравоохранения (ВОЗ) опубликовала свой первый перечень приоритетных педиатрических лекарственных форм антибиотиков как рекомендацию для научных исследований и разработки лекарственных форм антибиотиков, соответствующих возрасту. В перечне есть как азитромицин, так и нитрофурантоин. В настоящее время диспергируемые таблетки этих лекарственных препаратов отсутствуют или не имеют одобрения, а существующие жидкие формы неприятны на вкус и (или) содержат вспомогательные вещества, безопасность которых вызывает сомнения. Чтобы поддержать разработку соответствующих возрасту лекарственных форм этих двух антибиотиков, авторы предлагают целевые профили препаратов, созданные с применением методов ВОЗ. Для азитромицина оптимальной возрастной лекарственной формой и дозировкой являются диспергируемые таблетки 100 мг с риской или ородиспергируемая мультипартикулярная лекарственная форма, при этом минимальным требованием являются диспергируемые таблетки по 50 мг. Для нитрофурантоина оптимальной возрастной лекарственной формой и дозировкой являются ородиспергируемая мультипартикулярная лекарственная форма или диспергируемые таблетки с риской, при этом минимальным требованием являются диспергируемые таблетки. Исходя из рекомендуемой ВОЗ дозировки нитрофурантоина для детей в 4 мг/кг в день, оптимальной разовой дозой является 5 мг. Если возможно изготовление таблеток с риской, то следует разработать разовую дозировку в 10 мг для достижения гибкости лечения в различных педиатрических возрастных группах. Целью этих профилей является поддержка регулирующих органов, фармацевтических разработчиков, программ здравоохранения и других участников процесса в продвижении более безопасных, эффективных и пригодных для применения у детей антибиотических препаратов.

## Introduction

Bacterial infections continue to be among the main causes of death in children younger than 5 years globally.^1^ Despite the availability of several antibiotic classes, managing bacterial infections in children is challenging, particularly in low- and middle-income countries, where access to effective treatments and reliable microbiological diagnostics is often limited. These challenges are exacerbated by long-standing barriers to paediatric drug development, including limited financial incentives, low commercial interest and inadequate infrastructure for conducting clinical trials in children.^2^^–^^5^

One of the main problems for the treatment of children is the lack of age-appropriate formulations. This gap restricts therapeutic options, limits safe and effective use of the treatments available, and contributes to suboptimal care for children globally. In 2016, Member States of the World Health Organization (WHO) adopted resolution WHA69.20 to promote innovation and improve access to safe, effective, high-quality and affordable medicines for children.^6^ In response, WHO, together with its partners in the Global Accelerator for Paediatric Formulations Network, has strengthened its commitment to expanding the availability of age-appropriate formulations. A key initiative supporting this commitment is the paediatric drug optimization exercise, a strategic initiative designed to enhance the development and accessibility of age-appropriate formulations for children. The success of this initiative regarding treatments for human immunodeficiency virus, hepatitis C and tuberculosis underscores its potential to facilitate more rapid access to optimized treatments.^7^ To address the research gaps that limit access to suitable therapies for children with bacterial infections, WHO facilitated a paediatric drug optimization exercise to prioritize drugs for paediatric patients to ensure safe, effective and accessible antibiotic options are available for children.^8^

A key milestone in this effort was the release, in 2023, of WHO’s first priority list of paediatric antibiotic formulations.^8^ This list includes antibiotics already included in the *WHO Model list of essential medicines for children* and promising drugs in development. The list provides clear direction for future research and development and focuses attention on the specific needs of children and the need to ensure equitable access to safe, effective and age-appropriate antibiotic treatments worldwide.^8^

One of the priority antibiotics is azithromycin, a macrolide antibiotic. The WHO AWaRe (Access, Watch, Reserve) antibiotic publication recommends azithromycin as the first-choice drug for cholera, enteric fever, gonorrhoea, sexually transmitted infections caused by *Chlamydia trachomatis*, trachoma, yaws and chlamydia ophthalmia, and as the second choice for acute invasive bacterial diarrhoea.^9^ These conditions are important public health concerns.^9^^–^^11^ Additionally, azithromycin is one of the few choices for pertussis treatment. Among macrolides, azithromycin has several advantages, including a long half-life and a once-daily short-course regimen.^12^ Infections with macrolide-susceptible organisms are frequently treated with azithromycin. The antibiotic is widely available and relatively inexpensive, including in low- and middle-income countries. Azithromycin has been identified as a priority antibiotic because it can be used to treat a wide range of diseases, treatment durations are short, it is widely available and it has few adverse events.^8^

Another antibiotic on the priority list is nitrofurantoin, which belongs to the nitrofuran class. Nitrofurantoin is a legacy antibiotic that has been used to treat urinary tract infections for more than a decade. Urinary tract infections are among the most common bacterial infections affecting children. Nitrofurantoin has been included in the *WHO Model list of essential medicines for children*^10^ because it is effective for both treatment and prophylaxis of lower urinary tract infections in children.^10^ This antibiotic is especially valuable for children with genitourinary abnormalities who require prolonged prophylaxis, given its wide availability and affordability.^13^ Nitrofurantoin is highly effective against most Enterobacterales species responsible for urinary tract infections, including many extended-spectrum β-lactamase-producing strains.^8^ Due to its unique antibiotic class, nitrofurantoin shows limited cross-resistance with other antimicrobial agents. However, resistance has been reported in some multidrug-resistant pathogens.

In the AWaRe antibiotic book, nitrofurantoin is recommended as the first-choice drug for lower urinary tract infections.^9^ The WHO-recommended dosage of nitrofurantoin for treating urinary tract infections in children is 4 mg/kg per day, administered in two or four divided doses for 5 days.^9^^,^^10^ Prophylactic treatment is recommended for recurrent urinary tract infections. Nitrofurantoin is not recommended for more severe infections, such as upper urinary tract infections, because these life-threatening infections pose a risk of renal damage and require antibiotics that achieve adequate systemic concentrations, which nitrofurantoin does not provide.^9^

Nitrofurantoin can only be used after the neonatal period (at least 44 weeks of corrected gestational age) because of the risk of oxidative stress causing haemolytic anaemia in neonates with immature erythrocyte enzymes. The antibiotic has not been extensively studied in children younger than 12 years. Additionally, nitrofurantoin has been associated with haemolytic anaemia in individuals with glucose-6-phosphate dehydrogenase deficiency, necessitating caution in regions where this condition is prevalent.^14^

Currently, for both antibiotics, no dispersible tablet formulations are available, and the authorized liquid forms are not palatable and contain excipients that raise safety concerns, highlighting the need for improved paediatric formulations.

To support the development of age-appropriate formulations for these two antibiotics, we developed target product profiles using WHO’s established methods.^15^ These profiles aim to inform regulatory authorities, manufacturers, health programmes and other stakeholders about the need to develop optimum age-appropriate formulations of oral azithromycin and nitrofurantoin according to WHO recommendations. The comprehensive approach aims to ensure that the *WHO Model list of essential medicines for children* includes formulations that are truly suitable for children, addressing both technical and practical challenges faced in diverse health-care settings.^10^

## Methods

We reviewed available pharmacokinetic and dosing information, defining the ideal characteristics of the formulation and identifying key factors for its implementation, all aimed at speeding up the availability of child-friendly azithromycin and nitrofurantoin products. A WHO steering group, composed of 11 members from eight departments across four WHO divisions, was established to oversee this effort. The team responsible for drafting the target product profile included experts in science, clinical practice and public health, selected to reflect WHO’s commitment to geographic and gender balance, as well as diverse expertise.^16^

For each characteristic of the target product profile, product developers should aim to meet the optimum criteria whenever possible. Minimum criteria are given as a fallback if the preferred criteria are not feasible.

## Azithromycin

### Assessment of existing formulations

Based on a background assessment led by WHO and the global accelerator for paediatric formulations network,^17^ previously listed formulations of azithromycin, such as capsules, were found to be inappropriate for young children because of limited dose flexibility and acceptability issues. Although a powder for oral liquid formulation exists, excipient safety and reconstitution requirements are problematic in low- and middle-income settings. Thus, an age-appropriate oral formulation of azithromycin that includes safer excipients and does not require reconstitution is needed. Dispersible preparations exist in India, which suggests the potential feasibility of developing dispersible tablets; however, these preparations are not registered or marketed in other territories and jurisdictions.

### Target product profile^16^

#### Indication for use (compulsory)

The product should target specific indications, ideally covering chlamydial ophthalmia, trachoma, cholera, dysentery, enteric fever, pertussis, yaws, scrub typhus and atypical pneumonia. At a minimum, the product should target trachoma, cholera, yaws, pertussis and enteric fever.

#### Target population (compulsory)

The target population includes children weighing between 2.5 kg and 25.0 kg.

#### Safety

The active pharmaceutical ingredients should have a safety profile supported by evidence of bioequivalence, while excipients should meet regulatory requirements for inactive ingredients.^18^

#### Efficacy

Efficacy should be shown by establishing bioequivalence with the reference product in human studies.

#### Pharmaceutical form

Since the indications for azithromycin use span the whole age spectrum, we identified the need for formulations suitable for neonates, infants and children who are unable to swallow tablets, without compromising shelf life and other logistical considerations (for example, transport and ease of storage). We also considered orodispersible multiparticulate formulations (for example, sprinkles, minitablets and granules), nasal formulations and oral films, in addition to dispersible tablets. However, issues with drug loading for nasal and oral films limited their applicability.

Thus, orodispersible multiparticulate formulations and (functionally) scored dispersible tablets are the optimum formulations to be developed. Dispersible tablets are the minimum standard. If the development of functionally scored dispersible tablets is pursued, equal distribution of active pharmaceutical ingredients should be ensured.

#### Unit dose

Optimum dosing for various indications of azithromycin ranges from 10 mg to 20 mg per kg of body weight. We identified single scoring of dispersible tablets as the easiest form to break for usage, without requiring long-term storage of the remaining pieces. We considered and modelled various dispersible tablet sizes, including 60 mg, 80 mg, 100 mg and 120 mg. Since a single formulation at 60 mg would require five tablets for children weighing 15 kg or more, we concluded that the availability of functionally scored dispersible tablets sized at 100 mg would reduce the number of tablets to be given to older children, while being compatible with weight-band dosing. We also considered the option of double scoring. However, double scoring has several challenges, particularly related to achieving uniform distribution of the active pharmaceutical ingredient and difficulties associated with handling and storing tablet fragments.

Therefore, if orodispersible multiparticulate formulations are developed, or if scoring of dispersible tablets is not feasible, 50 mg should be the dosage strength.

#### Weight-based dosing

The dosage form should align with WHO weight-band dosing, ideally allowing use across multiple weight bands.

#### Size of the dosage form

The size of the dosage form should be adapted for children. Multiparticulate formulations should conform with standard size guidelines, and dispersible tablets should require only minimal liquid to form a homogeneous dispersion.

#### Acceptability and palatability

Palatability is essential for optimum formulations for use in children. Formulations should have a child-friendly, taste-masked flavour and good mouthfeel, confirmed through acceptability studies. At a minimum, taste and mouthfeel should be acceptable through the use of excipients, particularly flavours and sweeteners, commonly used in paediatric formulations.

#### Administration

Administration should be straightforward, requiring minimal manipulation by caregivers and reducing opportunities for medication refusal. If supplied in bottles, packaging should have a child-resistant cap.

#### Administration device

For the optimum formulation, no administration device should be required. If a device is unavoidable, such as a dosing cup or spoon, the minimum requirement is that it comes with simple, easy-to-follow instructions for use, suitable for people with low literacy.

#### Preparation before administration

In the optimum formulation, the product should not require multistep preparation by the end-user before administration. The antibiotic should be easy to prepare, for example, by mixing with water, milk or food, and should have clear instructions suitable for people with low literacy. At minimum, the product should be easy to prepare with water, milk or food, with clear instructions suitable for people with low literacy.

#### Stability and storage

The optimum formulation should be suitable for all climatic zones, including Zone IVb of the International Council for Harmonisation of Technical Requirements for Pharmaceuticals for Human Use (that is, 30 °C and 75% relative humidity).^19^ The antibiotic should have a total shelf life of at least 24 months and should not require special transport, storage or cold chain. At minimum, the product must be able to be transported and stored easily within the supply chain, and be stored by the end-user with no cold chain needs.

#### Packaging

The packaging should be compact, lightweight, easy-to-open and administer, inexpensive, sustainable (that is, designed, produced, and disposed of in ways that minimize its environmental impact while maintaining product safety and functionality), cost-effective to transport and child-proof.

#### Cost

For the optimum formulation, the total cost of goods and landed costs should be the same as, or lower than, existing formulations. The minimum requirement permits additional costs compared with existing formulations, provided they remain acceptable and affordable to caregivers, programme managers and funders.

#### Regulation

The optimum formulation should have a defined registration plan that follows reliable practices and seeks global registration whenever possible. At minimum, regulatory requirements in the intended end-user countries should be addressed from the outset.

#### Disability requirements for product label

Both optimum and minimum formulations should consider end-user disabilities; the optimum version should include accessibility features such as braille labelling or so-called talking patient information.

## Nitrofurantoin

### Assessment of existing formulations

The main problems with available formulations of nitrofurantoin are poor dose flexibility and the frequency of administration (four times a day). The available oral solid dosage form, 100 mg, is unsuitable for delivering an appropriate dose to children younger than 12 years.^13^ A 50-mg dose tablet preparation was therefore added to the WHO *Model list of essential medicines for children* in 2023.^10^ The oral liquid form (25 mg per 5 mL) is needed for children who are unable to swallow tablets; however, it is not palatable, contains excipients of concern and has a short shelf life once opened. The taste-masking in current formulations is inadequate to overcome palatability issues.

### Target product profile^16^

#### Indication for use (compulsory)

The product is intended for prophylaxis of urinary tract infections and the treatment of lower urinary tract infections, in accordance with WHO guidelines.

#### Target population (compulsory)

The target population includes children from 4 weeks of age (in case of preterm newborns, infants with at least 44 weeks of corrected gestational age) up to 35 kg in weight.

#### Safety

The active pharmaceutical ingredients should have a safety profile supported by evidence of bioequivalence, while excipients should meet regulatory requirements for inactive ingredients.^18^

#### Efficacy

Efficacy needs to be shown by establishing bioequivalence with the reference product in human studies.

#### Pharmaceutical form

The optimum pharmaceutical form is orodispersible multiparticulate formulations, such as minitablets or sprinkles. Functionally scored dispersible tablets or dispersible tablets are an acceptable minimum. The availability of dispersible scored tablets (where the taste remains masked after breakage) or dispersible multiparticulate formulations would overcome most of the existing challenges with current formulations, including the challenges related to excipients and packaging in glass bottles.

#### Unit dose

Reducing the strength of the dosage form would remove the need to break tablets to deliver smaller doses to young children. To optimize dosing, smaller dosage strengths are needed, for example, lower than 50 mg and 100 mg. Based on background modelling, 5 mg increments are required to deliver appropriate dosing across the weight spectrum. Thus, if orodispersible multiparticulate formulations are developed, or if scoring of dispersible tablets is not feasible, 5 mg should be the dosage strength.

A 10 mg tablet is the minimum dosage strength when a scored dispersible tablet is developed.

#### Weight-based dosing

The dosage form should align with WHO weight-band dosing, ideally allowing use across multiple weight bands.

#### Size of the dosage form

The size of the dosage form should be adapted for children. Multiparticulate formulations should conform with standard size guidelines, and dispersible tablets should require only minimal liquid to form a homogeneous dispersion. 

#### Acceptability and palatability

Palatability is essential for optimum formulations for use in children. Formulations should have a child-friendly, taste-masked flavour and good mouthfeel, confirmed through acceptability studies. At a minimum, taste and mouthfeel should be acceptable through the use of excipients, particularly flavours and sweeteners, commonly used in paediatric formulations.

#### Administration

Administration should be straightforward, requiring minimal manipulation by caregivers and reducing opportunities for medication refusal. If supplied in bottles, packaging should have a child-resistant cap.

#### Administration device

For the optimum formulation, no administration device should be required. If a device is unavoidable, such as a dosing cup or spoon, the minimum requirement is that it comes with simple, easy-to-follow instructions for use, suitable for people with low literacy.

#### Preparation before administration

In the optimum formulation, the product should not require multistep preparation by the end-user before administration. The antibiotic should be easy to prepare, for example, by mixing with water, milk or food, and should have clear instructions suitable for people with low literacy. At minimum, the product must allow for simple preparation with clear instructions.

#### Stability and storage

The optimum formulation should be suitable for all climatic zones, including Zone IVb of the International Council for Harmonisation of Technical Requirements for Pharmaceuticals for Human Use (that is, 30 °C and 75% relative humidity).^19^ The antibiotic should have a minimum shelf life of at least 24 months and should not require special transport, storage or cold chain. At minimum, the product must be able to be transported and stored easily within the supply chain, and be stored by the end-user with no cold chain needs.

#### Packaging

The optimum packaging should be compact, lightweight, easy-to-open, inexpensive, sustainable, simple to administer, cost-effective to transport and child-proof.

#### Cost

For the optimum formulation, the total cost of goods and landed costs should be the same as, or lower than, existing formulations. The minimum requirement permits additional costs compared with existing formulations, provided they remain acceptable and affordable for caregivers, programme managers and funders.

#### Regulations

The optimum formulation should have a defined registration plan that follows reliable practices and seeks global registration whenever possible. At minimum, regulatory requirements in the intended end-user countries should be addressed from the outset.

#### Disability requirements for product label

Both optimum and minimum formulations should consider end-user disabilities; the optimum version should include accessibility features such as Braille labelling or so-called talking patient information.

## Discussion

Ensuring access to new and essential antibacterial therapies is a fundamental aspect of achieving universal health coverage. The comprehensive approach of target product profiles for age-appropriate formulations aims to ensure that the WHO *Model list of essential medicines for children* includes formulations that are truly suitable for children, addressing both technical and practical challenges faced in diverse health-care settings. Developers are encouraged to implement access and stewardship plans that guarantee the availability of paediatric formulations at affordable prices. To support global access, developers should collaborate with WHO and global accelerator for paediatric formulations network partners where relevant.

Responsible use of antibiotics and antibiotic stewardship programmes are essential to preserving the efficacy of new antibacterial agents. Developers should not register these products for use in animals or plants, or create similar treatments for use in these areas. The access and stewardship plan should be grounded in ethical promotion and distribution practices. Additionally, manufacturing should adhere to best industry practices for managing environmental emissions to reduce the risk of spreading antimicrobial resistance.
